# A word embedding technique for sentiment analysis of social media to understand the relationship between Islamophobic incidents and media portrayal of Muslim communities

**DOI:** 10.7717/peerj-cs.838

**Published:** 2022-01-31

**Authors:** Ishfaq Ali, Muhammad Asif, Isma Hamid, Muhammad Umer Sarwar, Fakhri Alam Khan, Yazeed Ghadi

**Affiliations:** 1 Department of Computer Science, National Textile University, Faisalabad, Punjab, Pakistan; 2 Department of Computer Science, Government College University, Faisalabad, Punjab, Pakistan; 3 Department of Information and Computer Science, King Fahd University of Petroleum and Minerals, Dhahran, Saudi Arabia; 4 Department of Software Engineering/Computer Science, Al Ain University, Al Ain, UAE

**Keywords:** Computer aided design, Mobile and ubiquitous computing, Islamophobic, News stories, Sentiment analysis, Natural language processing

## Abstract

Islamophobia is a sentiment against the Muslim community; recently, atrocities towards Muslim communities witnessed this sentiment globally. This research investigates the correlation between how news stories covered by mainstream news channels impede the hate speech/Islamophobic sentiment. To examine the objective mentioned above, we shortlisted thirteen mainstream news channels and the ten most widely reported Islamophobic incidents across the globe for experimentation. Transcripts of the news stories are scraped along with their comments, likes, dislikes, and recommended videos as the users’ responses. We used a word embedding technique for sentiment analysis, *e.g*., Islamophobic or not, three textual variables, video titles, video transcripts, and comments. This sentiment analysis helped to compute metric variables. The I-score represents the extent of portrayals of Muslims in a particular news story. The next step is to calculate the canonical correlation between video transcripts and their respective responses, explaining the relationship between news portrayal and hate speech. This study provides empirical evidence of how news stories can promote Islamophobic sentiments and eventually atrocities towards Muslim communities. It also provides the implicit impact of reporting news stories that may impact hate speech and crime against specific communities.

## Introduction

According to the Merriam-Webster dictionary, the term “phobia” is an exaggerated, inexplicable, and illogical fear toward a particular entity or object or their classes (Social Network, 2020). It is hard to examine individual tendencies (personal reasons). The source of such is hard to determine; however, it exists among individuals or groups of people. The definition of Islamophobia is an exaggerated fear, hostility towards Islam and the Muslim community (Mehdi, 2017). Islamophobic marginalization results in bias, discrimination, and the marginalization and exclusion of Muslims from social, political, and civic life. Although it is believed 9/11 was a cause of Islamophobia, it has existed in different parts of the world since the inception of the Muslim empire (crusades, Spanish hate about Moors, and Indian people).

Al Jazeera, a leading news channel, summarized recent violent attacks against the Islamic community in different parts of the world, following a rise in frequency. Their summary, along with work by (Every-Palmer et al., 2020), mentions an attack on two mosques in New Zealand, killing 51 people and other attacks in the UK, USA, and several more countries. The Muslim genocide was also reported, which killed thousands of Muslims and compelled millions more to migrate. There is a list of recent violent attacks in the USA (Hafez, 2020), and according to Statista, anti-Muslim attacks have been on the increase since 9/11 (Statista, 2020).

Islamophobia is an evolving Social Science comparative phenomenon. Yet no generally accepted Islamophobia concept allows for systematic comparative and casual study (Edelmann et al., 2020). Anti-Muslim sentiments date back to the fall of the Roman Empire reached their peak during the crusades. These sentiments were also witnessed during Ottoman rule and a recent wave of anti-the Muslim sentiment in late 1990, especially after 9/11 (ResearchGate, 2020). Islamophobia is a matter of social sciences debate about how Muslims and their tradition affect other peoples’ lifestyles. Islamophobia and anti-Muslim sentiments have different grounds in the different regions, including misconceptions and historical grievance toward Muslim invaders. The recent wave of Islamophobia is caused by hate speech spread through either social media or mainstream media (Civila, Romero-Rodríguez & Civila, 2020). Fake news and manipulation of historical events float on social media even covered by mainstream media, somehow promoting Islamophobia, eventually causing violent incidents against the Muslim community across the globe (Saeed, 2007; Gottschalk, Greenberg & Greenberg, 2008; Qian, 2019).

In today’s digitalized world, a person receives information at a higher pace about any incidents and develops mass sentiments about specific communities. This research paper has examined how reporting certain events develops public sentiments about the Muslim community. For this purpose, we have taken fourteen mainstream news channels reporting about incidents in the near past. We captured their YouTube stories transcripts and their public response. (in terms of likes, dislikes, and comments). This research activity’s objective is (a) the correlation of hate speech between the news story’s contents and its response to specific news channels. (b) Identification of news channels, information and their respective audience with a relatively higher number of hate words (promoting Islamophobia). The third objective is to understand the media’s impact on public sentiments toward Muslims.

This research activity’s essence is the sentiment analysis of YouTube videos’ transcripts and their responses (likes, dislikes, and comments). Data is gathered from YouTube using Data Miner’s customized recipe. To clean different activities for cleaning data has been carried out discussed in the subsection of methodology. Our *corpus* is English (We have used English channels only, and comments in other languages are translated into English); therefore, it is not multilingual sentiment analysis. We have used a bag of words models and specialized scales for sentiment analysis for hate words. Finally, canonical correlation is measured among incidents reported by various research news channels. The contribution of this research work is to simulate a state-of-the-art method for *corpus* collection from online videos and their respective responses from social media platforms. Furthermore, it also provides a method for sentiment analysis from multilingual data. It also provides empirical evidence regarding news portrayals’ impact upon hate speech.

“Literature Review” provides a literature review about the approaches used for data collection, sentiment analysis. “Methodology” provides a detailed overview of how *corpus* was collected; organized and canonical correlation was performed. Finally, “Discussion” concludes the article with a comprehensive discussion.

## Literature review

Terman (2017) investigated the correlation between the way the media portrays Muslim women and Islamophobia based on gendered orientalism. They utilized computational text analysis and modeled news media reports about Muslim women. Further study has been carried out by Cheshmedzhieva-Stoycheva (2018) about the portrayal of Bulgarian Muslims by British media. Another study was carried out by Mahmood & Salim (2020), investigating portrayal of Muslims on Facebook and other conventional US media. There are concerns that the US media portray the Middle East as a hotbed of extremists and fundamentalists, and these concerns were examined in detail Al Sharafat (2019). Velasco (2020) argue the significance of media, either conventional or modern platforms with a global audience over the internet/social media, in shaping cultures around the world, and particularly shaping views on Muslim communities. Critical portrayals of Muslims is not only an issue in USA/European media, but also in China, where Muslims are persecuted and Islamophobia is hosted on social media platforms (Luqiu & Yang, 2018; Raza, 2019; Luqiu & Yang, 2019); Chinese state media (either print or electronic media) portrays Muslims as the route cause of the Islamophobic sentiments in Chinese. The situation in the South Asian region is not different from the rest of the world, especially in the Indian media (Mukherjee, 2020; Sanjeev Kumar, 2016; Ireton & Posetti, 2018; I. T.-S. A. M. Academic and Undefined, 2007). Incidents of atrocities towards Muslim communities in India are numerous (Copsey et al., 2013; Batool, 2018).

The media have a significant role in shaping domestic and geopolitical landscapes. Approaches to determining the relation between media portrayals of Muslim communities with Islamophobic sentiments emergence utilized text analysis more than any other approach (up to 63% out of 38 studies). Text analysis provides sentiment analysis, along with many statistical measures, its utility in said domain more than any other approach. Our approach to understanding the correlation between portrayals for Muslim communities and Islamophobic sentiments in localities/demographics. These Islamophobic sentiments eventually cause atrocities towards Muslim communities. Muslim communities’ fear and hostility are also caused by Islamophobic sentiments (and media has a role in such sentiment development).

An approach (Hussain et al., 2021) harvested consumer sentiment using the HaaS crawler and then analyzed them using the AHP-IOWA method. The approach enables the decision-maker to address the nonlinear relationship of selection criteria. In Raza, Hussain & Merigó (2021), the researchers analyzed sentiment using deep learning methods. The authors compared RNN, LSTM and GRU approaches on cloud consumer sentiment and found that GRU outperforms all other methods.

Text analysis approaches for sentiment analysis (Khoo & Johnkhan, 2018; Asif et al., 2020; Krishna, Zambreno & Krishnan, 2013; Martins & Gomes, 2018) for political and social issues are coming under the umbrella of a discipline known as “Computational Sociology.” Computational sociology or computational social science is a research discipline that interfaces with computational and sociological disciplines (Edelmann et al., 2020). We may conclude the genre of the proposed work is a computational social science; therefore, the approach for conducting this research is similar to social sciences research projects (Haider et al., 2021). A detail of the social science research process is given in the Background subsection.

The objective of the research paper is to understand the role of anti-Muslim sentiment from news story portrayals of the Muslim community in mainstream media.

## Methodology

### *Corpus* collection

Carrying out a text mining-based project began with formulating a question followed by data collection and its cleaning, finally, its visualization (to inspect quality and type of data). The *corpus* creation step is crucial, *i.e*., representation for statistical or Machine learning modeling for several tasks, including sentimental analysis. Since our study is related to sentiment analysis, a generic overview of the text mining approach for sentiment analysis is as follows:

*Corpus* creation is a multistep process, begins with the decision of violent incidents globally that happened very recently. The list of recently happened events under consideration is shown in Table 1.

**Table 1:** Incident relevant data capturing.

Incidents	Geography
Delhi riots 2019	India South Asia
Myanmar genocide	Myanmar South East Asia
Citizen act of India	India South Asia
New Zealand Mosque attack	New Zealand
Australia veiled women attack	Australia
London knife attack	England
New York attack on Sikhs (as they were the Muslims)	USA
European report on Islamophobia related attacks	Different European countries
Syrian migration to Germany and other Western countries	Syria and Western countries
COVID-19 association with the Muslim	England and India and some other regions

**Figure 1:**
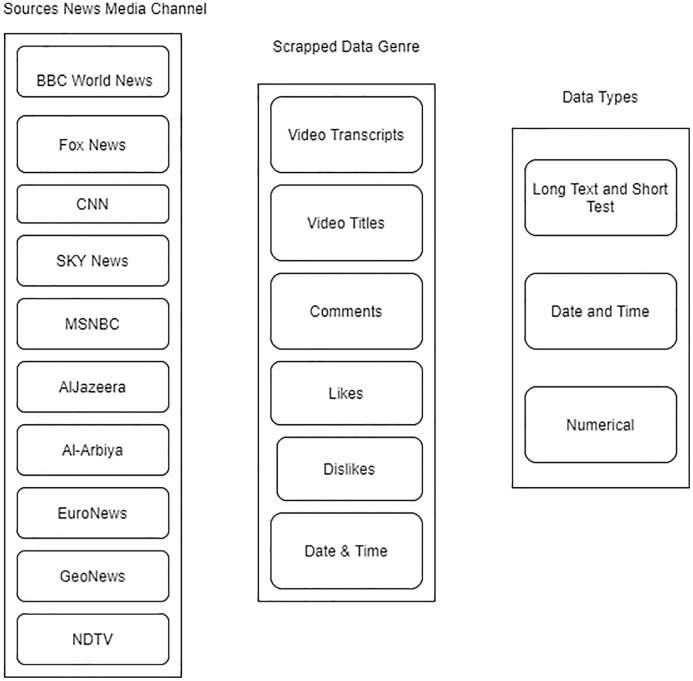
Data capturing approach.

Although some incidents in the list are not related to essentially Islamophobia, their respective response is Islamophobians. The respective news channels from which their information is captured are given in Fig. 1.

After deciding where and what to capture, the *corpus* creation's very first step is getting social media data using the R “vosonSML” library (CRAN-Package vosonSML, 2020) with the help of YouTube API. The final structure of the dataset, as captured from YouTube, is given in Table 2.

**Table 2:** Structure of dataset.

Table name	Description
Video_Transcript (Video_id, channel_id, Title, Text)	Table of transcripts,
Channels_data (channel_id, No_of_Subscriber, No_of_video)	Detail of each channel saved in this table
Video_Response (Video_id, no_of_Views, no_of_likes, No_of_Dislikes)	Responses other comments are saved in this table
Comments (Channel_id, Video_id, comet)	Comments in textual
Revive (Video_id, Titles)	Video Titles

The next step is its cleaning; we have done it using the “tm” library, including the number of activities for cleaning data. However, the following generic data cleaning pattern will be enough for social media data since we have multilingual data and a better result.

We manipulate the YouTube recommended algorithm to identify how other recommended video titles show Islamophobia related or not. The critical aspect of the YouTube recommendation algorithm somehow clusters videos of similar types. The algorithm for video recommendation is influenced by many factors such as user history for YouTube, locality, subscriptions of channels, videos, *etc*. However, all these factors can be reduced using Google Chrome or any other browser (new installation without login), or someone can use incognito/private mode to search for videos.

Another aspect of YouTube is three sorts of responses: Likes, Dislikes, and Comments (precisely, Likes and Dislikes did not help much but are still user responses). Although it’s not a valid argument, we can consider it Dislike as hate and Likes as appreciation. But how we can differentiate the Likes and Dislikes meant to be hate and gratitude is just an individual understanding prospect. Therefore, we have taken both with their meaning as the ratio of Dislikes/Likes videos. The user comments being the most contributing modality is scrapped using vosonSML (R-Library) and classified using Multilingual-Lexicon based sentence classification.

The final dataset consists of 38 videos, 1,018,500 comments, and 496 recommended video titles.

### Tools and resources

**Figure 2:**
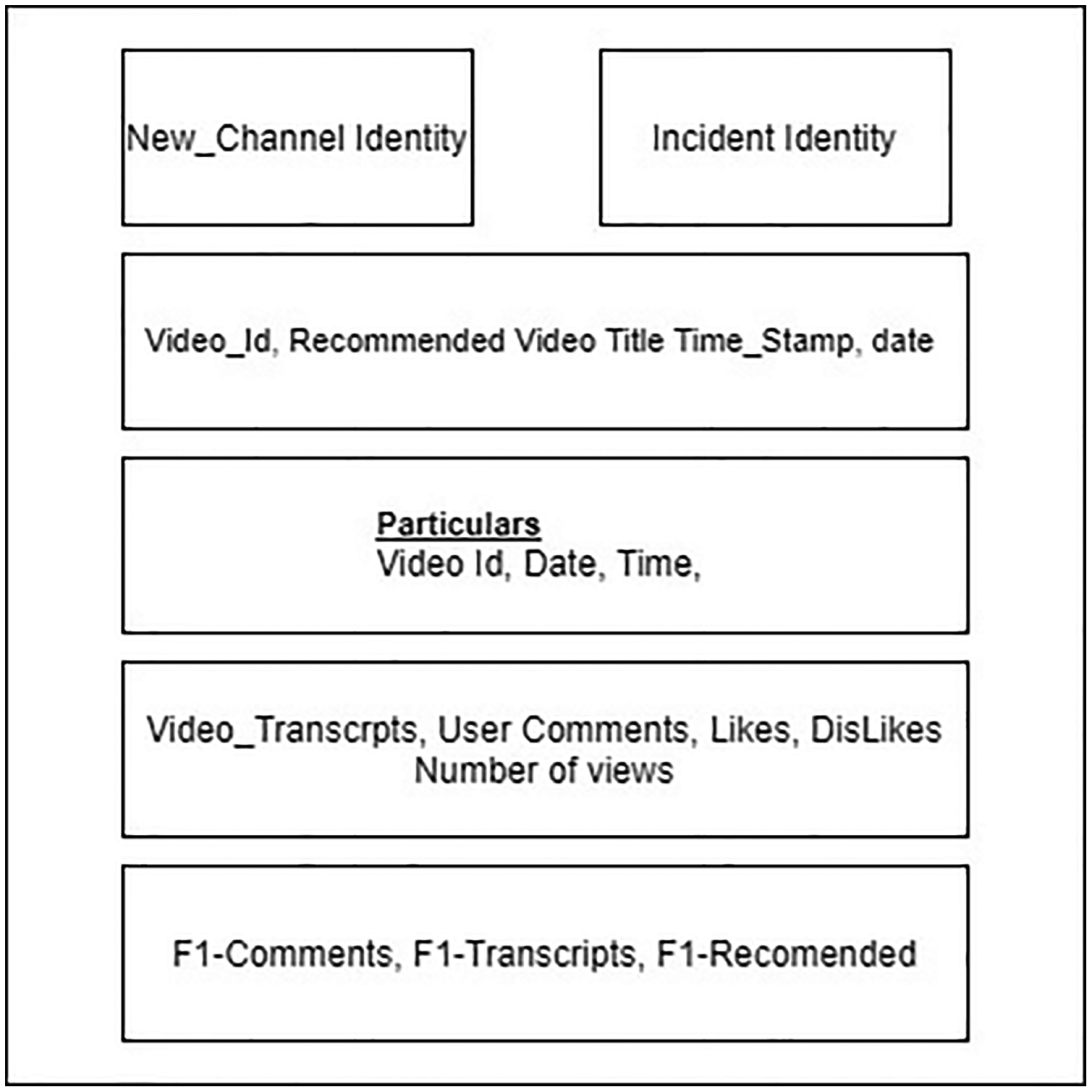
Aspects of data compilation.

The most significant resource is YouTube, and other resources are the website from where we have captured 40,800 Islamophobic and hate speech keywords for lexicon development. Another resource is lexicon development key words resource—. Some tools are used R (“tm”, “VosonSml”, “shinyApp”, “TubeR”). These libraries and web scrappers as “scrapper.io” and video transcripts captured manually scrapped, and finally, dataset architecture is given in Fig. 2. The dataset format includes six columns video_id, Likes, Dislikes, No_of_views, Video_transcript, and comments.

### Lexicon development for islamophobic words

Some examples of keywords: Islamic terrorism, Islamic fanatics, Islamic fundamentalism, Islamic extremists, Islamic radicals, Islamic fascists, Islamic fundamentalism, Islamists, jihadist, Islamism, militant Islam, radical Islam, political Islam, fanatical Islam, Islamo fascists, militant Muslims, and the Muslim terrorists.

### Classification of comments

Comments are classified using an R-Library tubeR (widely used for sentiment analysis), and the classification is carried out collectively on the entire repository and particular video. This illustrates the extent of hate speech in a specific video. It also enables cluster analyses of certain incidents that trigger Islamophobic sentiment. Moreover, there is some sort of similarity among the entire sentiment.

#### Video transcripts analysis

We have scraped video transcripts manually and performed sentiment analysis using the lexicon-based technique. A lexicon is prepared using terminologies widely used for hate speech or Islamophobic terms used for the Muslim community. We captured these terminologies from three different resources. However, sentences are not as structured as the standard text sentence (since these videos are transcripts of spoken words). These videos, however, are in the English language, therefore unilingual lexicon analysis.

#### Likes/dislikes ratio

Both Likes and Dislikes are user responses, having significant meaning (most YouTube users tend to click on the Like/Dislike button instead of commenting on a sentence). However, it is challenging to cultivate its true meaning in which users have used these buttons. For example, a violent incident against the Muslim community news story liked by the user meant to appreciate the event or its reporting and Dislike meant to protest the event or its reporting vice versa is also possible. Moreover, these buttons might be clicked accidentally or unnoticed act. Therefore, we devised a ratio to get public sentiment out of it, *i.e*., Like/Dislike and Dislike/Like with their alternative meaning (both positive and negative alternatively). These ratios provide input for overall analysis.

#### Recommended videos titles

YouTube video recommendations algorithm manipulation for capturing recommended videos. Video titles are also classified to learn the video under consideration is recommended Islamophobic or hate speech videos. The recommended videos are scraped by using a scraping tool, Webscrapper.io. To avoid YouTube video recommender influenced by user history and location, we have used chrome, which was newly installed without any user login. To verify these results, one should do the same. Those titles were not only considered for learning either the video is clustered with hate speech videos are Islamophobic videos (which is determined by their titles sentiment analysis) but also for current video types.

### Sentiment analysis

Sentiment analysis has a broader range of applications from business, social, political, medical, and scientific research. Sentimental analysis (a term used by business personal) or opinion meaning (the term used by academia) has many forms, such as opinion extraction, sentiment mining, or subject analysis (Aljuaid et al., 2021). Sentiment analysis studies views, opinions, attitudes, feelings, and personal assessments about a sure thing, environment, or other things. It broadly deals with individuals’ and groups’ sentiments about someone and something, *i.e*., products, facilities, societies, events, topics, and their attributes. The method used for the classification of textual data is as follows.

#### Data under consideration

1. Titles of videos in *corpus*
2. Titles of recommended videos
3. Sentences of video transcripts
4. Comments

#### Lexicon based sentiment

**Figure 3:**
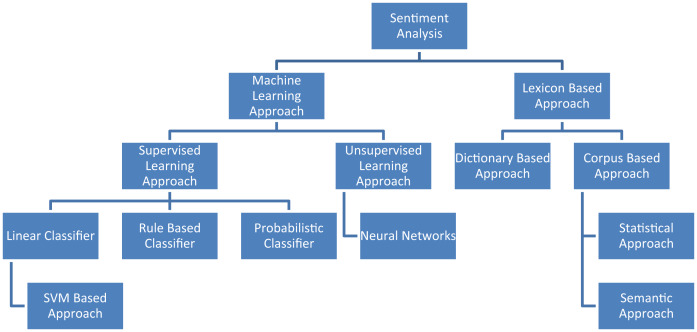
Different approaches to sentiment analysis.

Sentiment analysis refers to the use of natural language processing, text analysis, computational linguistics, and biometrics to systematically identify, extract, quantify, and study affective states and subjective information (also known as opinion mining or emotion; Rajput & Solanki, 2016). Different approaches to sentiment analysis, as shown in the following Fig. 3.

#### *Corpus*-based sentiment analysis techniques

“*Corpus*” can be defined as an electronically stored collection of spoken or written words. Authors identified critical characteristics of the *corpus* as machine-readable, authentic, representative of a particular language, or even multiple languages. *Corpus*-based lexicon analysis is a widely researched area for linguistics and relevant disciplines. However, its context-oriented utility was rarely, machine learning approaches for sentiment analysis and text classification were used in a particular context.

A summary of the extraction process, as described in the following paragraph. The first step of data extraction from the number of websites for *corpus* collection is crucial to collect relevant, empirically enough evidence for classification. The quantity and quality of data scrapped are measures to ensure the machine learning model’s accuracy. Multiple approaches are available for data collection, and many social media platforms provide APIs for scrapping data and many other techniques for scrapping websites. The next step is preprocessing scrapped data, including normalization, *i.e*., noise removal (special characters, numbers, multilingual). Some of these actions include removing, non-English words, removing uniform resource locators (URLs), slang word translation, removing extra letters from words, and Stemming. The third step is sentiment identification, sentiment word identification (SWI), which is used by many applications for opinion mining. The following subsection provides the approach employed.

**Step 1. Compilation of data**

For the sake of sentiment analysis, we have used textual data, *i.e*., Titles, transcripts, recommended video titles, and comments, and we have organized all these textual data based on video ID for simplicity and identification.

**Step 2 Preprocessing**

**Figure 4:**
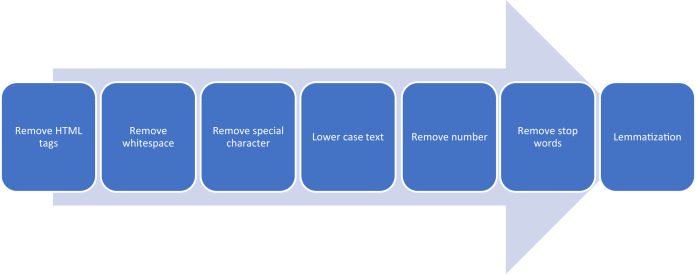
Noise removal from text.

Preprocessing of the text includes several activities, as shown in Fig. 4, for normalization. Moreover, another step was carried out to remove non-English comments from the user comments. The next thing we have done is to perform tokenization of words and implementation of TF-IDF model (term frequency-inverse document frequency) commonly used for natural language processing (NLP) tasks (Vijayarani, Ilamathi & Nithya, 2015). Our approach requires a word importance metric. TF-IDF Model will furnish it.

**Step 4. Sentiment identification**

Sentiment Identification is the key step for the identification of polarity. We have employed the sentiment identification technique (Yu, Deng & Li, 2020), *i.e*., polarity words. Their technique employs polarity identification without seed or any labeling showed significant results. The detail of this process, as given below.

**1. Word polarity vector**

The word polarity is either positive or negative and neutral. For each word in the document, the polarity is computed as follows

For each word ${c_{i\; }}\varepsilon \; corpus$ and its weight ${w_{i\; }}$from TF_IDF matrix. Their weight is computed as $\matrix{
   {{c_i}}  \cr 
   {{c_j}}  \cr 
   {{c_n}}  \cr 

 } $ multiplied with weight matrix as $\matrix{
   {{w_i}}  \cr 
   {{w_j}}  \cr 
   {{w_n}}  \cr 

 } $ to compute the final matrix. The polarity contains positive other case is negative

(1) $${{\bf{w}}_{\bf{i}}}\left( {{{\bf{c}}_{\bf{i}}}} \right).{{\bf{w}}_{\bf{j}}}\left( {{{\bf{c}}_{\bf{j}}}} \right) \ge 0$$

The next step is to compute document polarity by computing cosine polarity for each document, as given below (Raza et al., 2021)

(2) $${\rm d}\left( {\rm t} \right) = {\rm cosine}\displaystyle{{ft.\; w} \over \|w\|}$$

Finally, document polarity of the sentence provides an estimation of the polarity of the entire transcript of the video and entire comments. The polarity is calculated using a sentiment analysis scheme as described in Arnold (2017):

(3) $${c_i} = \left| {{y_i} - {l_i}} \right| = \left| {\displaystyle{{\vec f_i^T.\overrightarrow {\; w} } \over \|{\vec w}\|} - {l_i}} \right|$$

The final computation modules are described in Raza et al. (2021):

$$\mathop {\min }\limits_{\vec x} E\left( {\vec x} \right) = \mathop \sum \limits_{i - 1}^n {\left( {\vec f_i^T.\vec x - {l_i}} \right)^2}$$

(4) $${\rm Such\; that }\; \|\vec x\| = 1$$

**Figure 5:**
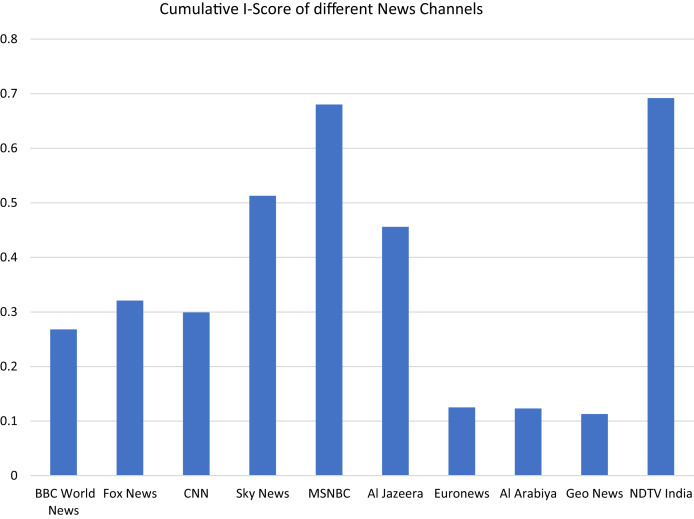
Cumulative I-Score of different news channels.

Experimentation was carried out using lexicon-based on MPQA (2020). The next step is to calculate the I-Score, which is defined over the document-polarity as follows (Brazdil, 2009):

(5) $${I_s} = \mathop \sum \limits_i^n \displaystyle{{cosine\left( {{f_i},\; {w_i}} \right)} \over \|w\|}$$

where *f_i_* is the frequency is the frequency of polar term and ${w_i}$ the weight of a particular term. Therefore, each video has its cumulative partiality score, a higher value of ${I_s}$ represents higher Islamophobic content in video and its responses. Figure 5 illustrates the I-0 score of different contents.

These I-Scores provide information about how some news channels portrayals impact Muslim communities across the globe with their direct impact, based on cumulative I_s_ transcripts as given in Fig. 5.

**Figure 6:**
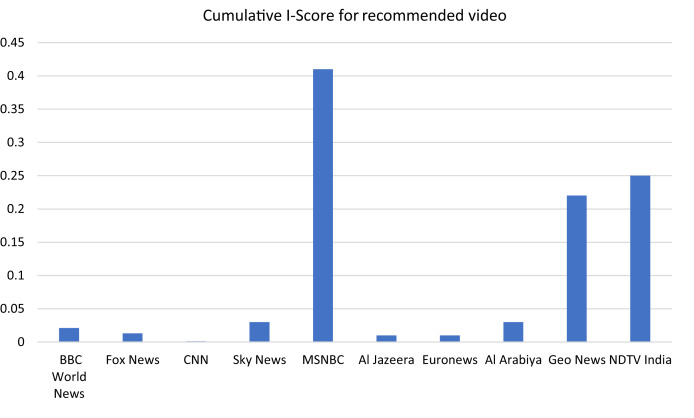
Cumulative I-Score for recommended videos.

Another measure that describes how YouTube video groups its video following its recommendations. Although many other factors impact YouTube video recommendations, we have used a chrome web browser without any login/user account. In addition, deleting all temporary files from the local machine and deleting history before scrapping videos from each channel minimize other influencing factors the recommendation. Figure 6 represents the cumulative I-Score of their respective recommended videos.

**Figure 7:**
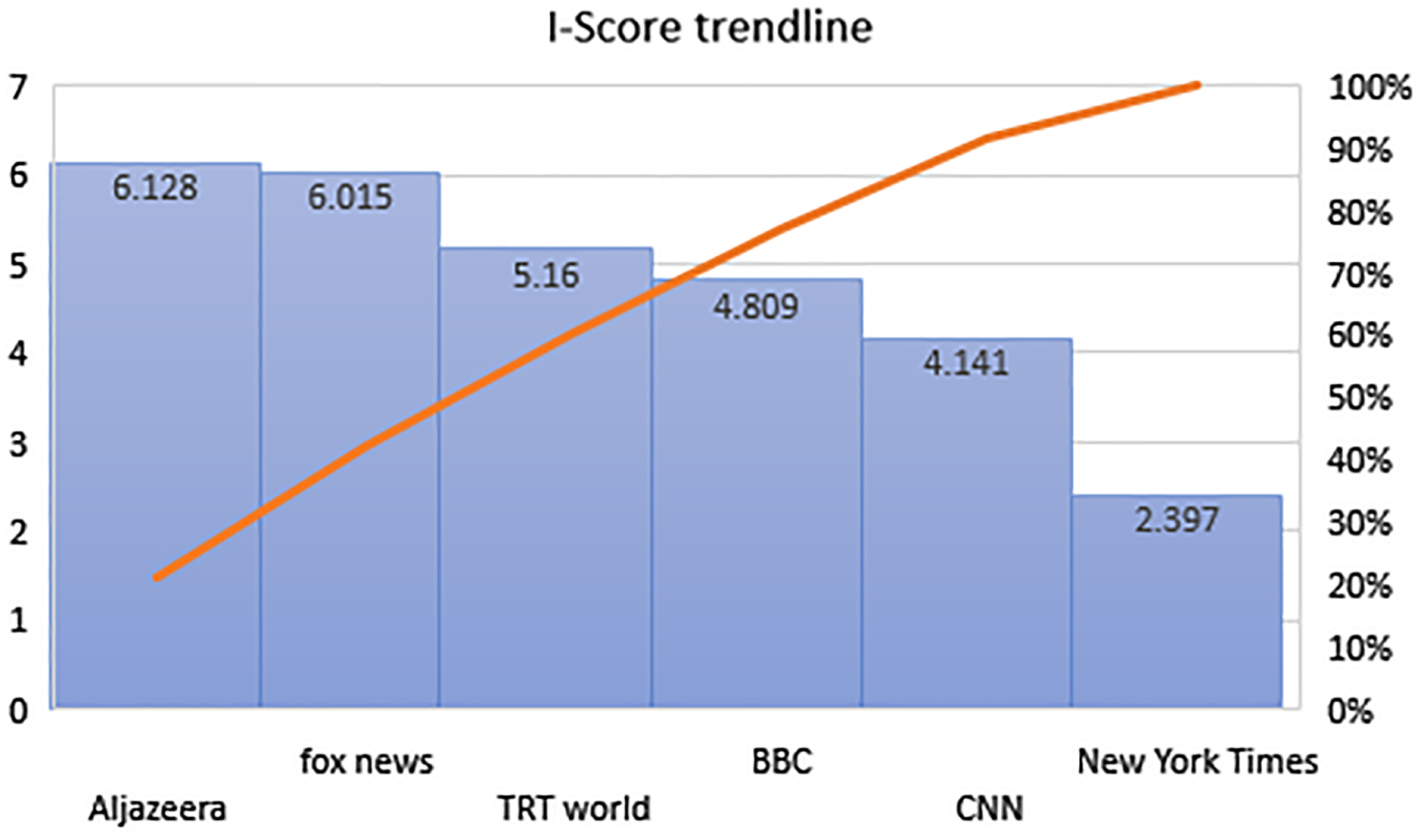
Trends in I-Score.

We have compared the cumulative I-Score for video transcripts and recommended video titles, as shown in Fig. 7.

However, the cumulative I-Score for comments is not compared because it depends on respective videos. Furthermore, these videos depend on incidents; therefore, relevant canonical correlation is performed to understand the entire content.

### Canonical correlation analysis CCA of data

**Figure 8:**
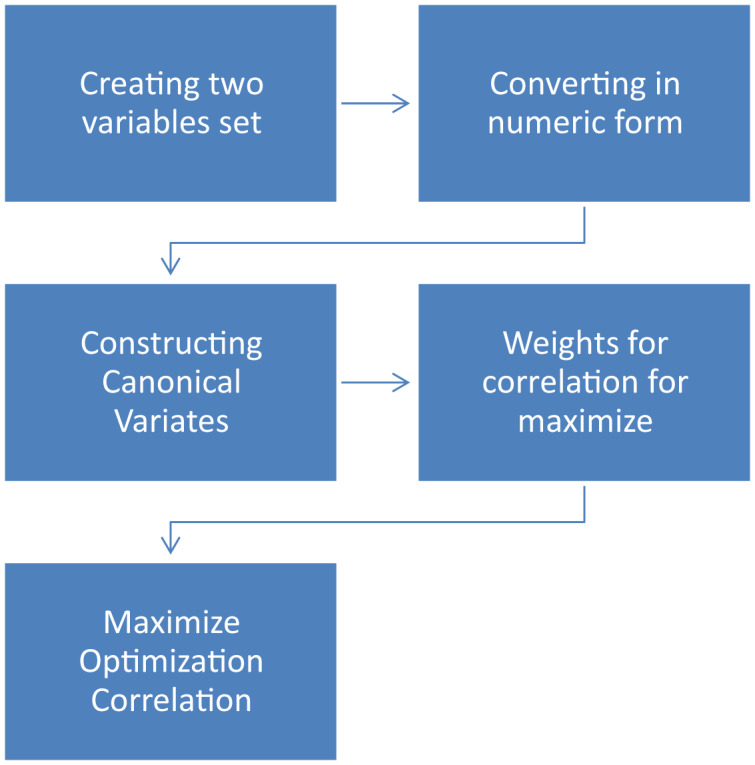
Canonical correlation process.

Canonical Correlation Analysis (CCA) (Thompson, 1984, 2005) is a statistical tool used to investigate relationships between two or more variable sets consisting of a minimum of two variables each (MPQA, 2020; Brazdil, 2009). Researchers prefer multivariate methods instead of univariate solutions for the following two reasons, *i.e*., it avoids Type I error or inflation of experimentation. Second, by considering all the variables simultaneously in a single analysis, CCA honors (Dattalo, 2014). The ecological truth of getting all the variables in nature. They can communicate with one another. In other words, the same thing Data can yield (a) statistically non-significant results when analyzed with multiple univariate methods and (b) sizes of zero consequence, but when diagnosed with CCA may deliver (a) statistically significant and statistically significant results. (b) giant impact sizes. And in such situations, we prefer to believe the multivariate findings since we assume that all the variables interact. For each other, and that is just an appraisal that honors. This possibility generalizes to the fact well, to reality—calculation of I-Score for each video and textual dimension, *i.e*., titles, comments, and transcripts. The Canonical Correlation Process is shown in Fig. 8.

This research’s primary objective is to learn the impact of news story coverage on Islamophobia on viewers’ point of view from their response. Therefore, we compile three types of user’s responses, *i.e*., Like, Dislike, and Comments, along with another construct, *i.e*., recommended videos. The YouTube video recommender is somehow an aggregated viewer response as the viewer tends to watch similar videos simultaneously. These four responses are compiled into a single spreadsheet for further analysis, as shown in Table 3.

**Table 3:** Dataset for canonical correlation.

Table name	Description
Video_Transcript (Video_id, channel_id, Text)	Table of transcripts,
Channels_data (channel_id, No_of_Subscriber, No_of_video)	Detail of each channel saved in this table
Video_Response (Video_id, no_of_Views, no_of_likes, No_of_Dislikes)	Responses other comments are saved in this table
Comments (Channel_id, Video_id, com_text)	Comments in textual

These responses struct utilized further to learn the correlation between user response and news story covered. The correlation analysis is carried out concerning incidents and concerning specific news channels. These correlation analyses are carried out in a simple spreadsheet, *i.e*., Microsoft Excel, using as a data analysis tool.

The dataset is bisected into set A contains Transcripts, Titles, recommended titles, *i.e*., Words of news channels, and Set B has comments, number of views, number of likes, and number of dislikes, *i.e*., user’s response, and our objective is to learn canonical correlation analysis CCA.

The next step is converting all textual data into a normalized form, *i.e*., metric form, to carry out canonical correlation, as shown in Table 4.

**Table 4:** F1 Scores of textual data.

Sr. No.	Video_id	F1 Comments	Likes	Dislikes	Like/Dislike	Dislike/Like	Video_recommender
1	4vtVEwbbvJI	0.67	744	3,939	0.188880427	5.294354839	Yes
2	ALFBSThw3FA	0.71	3,708	3,069	1.208211144	0.827669903	Yes
3	o_R8QJzU2ys	0.9	1,447	488	2.965163934	0.337249482	Yes
4	S0TpkSrWKI4	0.12	3,109	999	3.112112112	0.321325185	No
5	79Hr03bxzns	0.75	269	3,269	0.082288162	12.15241636	Yes
6	“-But7VUveAc”	0.15	3,315	4,156	0.797641963	1.253695324	Yes
7	DewWSGTwOXo	0.09	1,925	247	7.793522267	0.128311688	Yes
8	Qa9w3wUWWAE	0.69	2,834	1,104	2.567028986	0.389555399	No
9	4R5cpdUmmqU	0.32	633	967	0.654601861	1.52764613	Yes

The next step is computing correlation weights for correlation analysis as follows CS1 represents the correlation coefficient between video transcript and comments, CS2 is between video transcript and Likes/Dislikes, CS3 is between video transcript, and CS4 is between video transcript and its recommended videos. These four coefficients represent the correlation between all four constructs, as shown in Table 5.

**Table 5:** Four constructs of weights for CCA.

Sr. No.	Channel Id	CS-1	CS-2	CS-3	CS-4	CS-5
1	1	0.32	0.31	0.17	0.63	0.05
2	1	0.49	0.42	0.74	0.88	0.21
3	1	0.2	0.67	0.51	0.73	0.39
4	1	0.04	0.48	0.88	0.89	0.24
5	1	0.54	0.22	0.8	0.68	0.04
6	1	0.56	0.02	0.18	0.82	0.79
7	1	0.17	0.83	0.08	0.26	0.42
8	1	0.2	0.89	0.28	0.53	0.47
9	1	0.43	0.2	0.15	0.44	0.22

**Figure 9:**
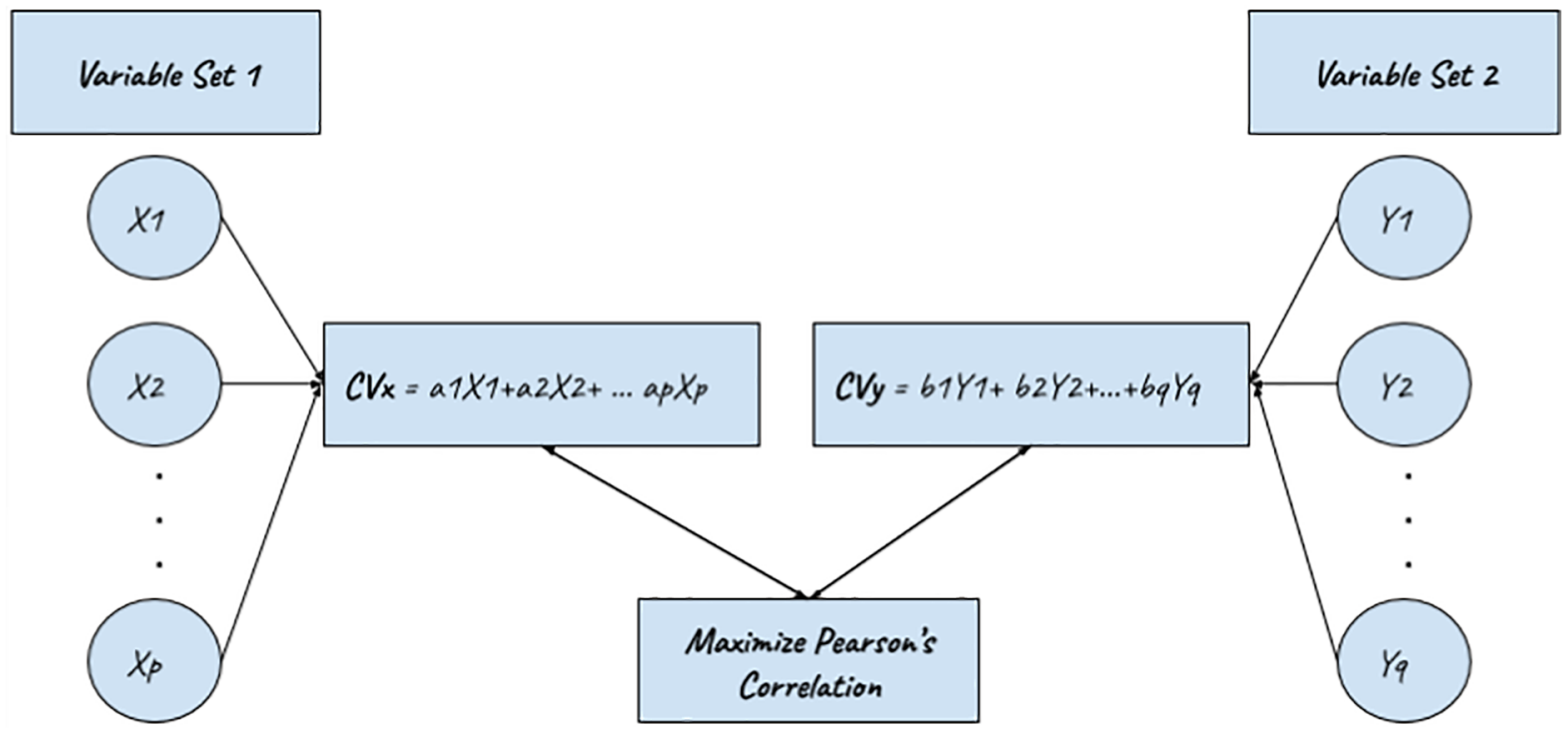
CCA process (Dattalo, 2014).

The fourth step is to compute Pearson correlation; for CCA, the entire process is depicted in Fig. 9, which is taken from (Dattalo, 2014).

The respective results of CCA for each news channel are shown in following Table 6. Entries in Table 6 are multiplied by 100 to make them more presentable.

**Table 6:** Incidents of correlation multiplied by 100.

News channel	Inc_1	Inc_2	Inc_3	Inc_4	Inc_5	Inc_6	Inc_7	Inc_8
BBC World News	▼ −38	▲ +53	▼ −20	▼ −1	▲ 33	▼ −8	▲ 44	▲ 36
Fox News	▲ 50	▲ +13	▼ −6	▼ −23	▲ 44	▼ −23	▲ 58	▼ −4
CNN	▲ 60	▲ 31	▲ 3	▼ −1	▼ −5	▲ 64	▲ 37	▲ 21
Sky News	▲ 42	▲ 8	▼ −1	▲ 19	▼ −10	▲ 1	▼ −20	▲ 23
MSNBC	▲ 54	▲ 63	▼ −1	▲ 64	▲ 10	▼ −12	▼ −6	▼ −19
Al Jazeera	▼ -13	▼ −16	▲ 60	▲ 47	▲ 48	▲ 24	▲ 12	▼ −9
Euronews	▲ 37	▲ 17	▲ 57	▲ 62	▼ −17	▲ 25	▲ 27	▲ 64
Al Arabiya	▲ 42	▼ −2	▲ 8	▼ −13	▲ 10	▲ 40	▲ 4	▲ 62
Geo News	▲ 22	▲ 4	▲ 34	▼ −7	▲ 50	▲ 47	▲ 17	▲ 4

## Discussion

Our approach is determined to furnish empirical evidence to demonstrate the relationship between news stories’ portrayal of Muslim communities. We have compiled ten news channel stories about ten global incidents. We collected a dataset from scratch, *i.e*., scrapping data from YouTube videos transcripts (News Stories) and their respective responses, *i.e*., comments, likes, dislikes, and the number of views. Another construct we considered is YouTube video recommendation (their titles are only under consideration). This dataset underwent different steps to eventually, all variables achieved homogeneous data types for final canonical correlation analysis. The reader carefully examines the encounter in many positive values of the correlation coefficient. This positivity of the coefficient reflects the relationship between news stories portrayals and Islamophobic sentiment; this phenomenon can also be visualized from Figs. 5 and 6.

This study provides a systematic approach for computational social sciences research problems as it provides a mechanism for empirical evidence for qualitative research questions. Since Islamophobic sentiment is a qualitative research area for diverse academic interests, including computational social sciences, it provides a comprehensive systematic approach to a social media analysis project. This research project portrays a multistep project for social media analysis for diverse research questions for several academic disciplines.

Although many other significant factors are affecting Islamophobic sentiment, *i.e*., historical influences, social and cultural differences, and even personal differences, Islamophobic sentiments arousal in certain parts of the world have their own particular social, economic, cultural, and geopolitical phenomenon, *i.e*., India, Myanmar, and China. However, the role of the news cannot be neglected.

## Disclaimer

This research aims to understand the correlation between Islamophobic sentiments with portrayals of Muslims across the globe. This study is neither targeting any community nor any institute.

## Supplemental Information

10.7717/peerj-cs.838/supp-1 Supplemental Information 1 Dataset and R code used in the project. The dataset was scraped from Youtube.

## Additional Information and Declarations

### Data Availability

The following information was supplied regarding data availability: The dataset used in the project was taken from YouTube stories and is available in the Supplemental File.
